# The CaMKII phosphorylation site Thr1604 in the Ca_V_1.2 channel is involved in pathological myocardial hypertrophy in rats

**DOI:** 10.1080/19336950.2020.1750189

**Published:** 2020-04-14

**Authors:** Jingyuan Li, Siqi Wang, Jie Zhang, Yan Liu, Xi Zheng, Fan Ding, Xuefei Sun, Meimi Zhao, Liying Hao

**Affiliations:** Department of Pharmaceutical Toxicology, School of Pharmacy, China Medical University, Shenyang, China

**Keywords:** Ca_V_1.2 channel, CaMKII, Thr1604, phosphorylation, myocardial hypertrophy

## Abstract

Residue Thr1604 in the Ca_V_1.2 channel is a Ca^2+^/calmodulin dependent protein kinase II (CaMKII) phosphorylation site, and its phosphorylation status maintains the basic activity of the channel. However, the role of Ca_V_1.2 phosphorylation at Thr1604 in myocardial hypertrophy is incompletely understood. Isoproterenol (ISO) was used to induce cardiomyocyte hypertrophy, and autocamtide-2-related inhibitory peptide (AIP) was added as a treatment. Rats in a myocardial hypertrophy development model were subcutaneously injected with ISO for two or three weeks. The heart and left ventricle weights, each of which were normalized to the body weight and cross-sectional area of the myocardial cells, were used to describe the degree of hypertrophy. Protein expression levels were detected by western blotting. CaMKII-induced Ca_V_1.2 (Thr1604) phosphorylation (p-Ca_V_1.2) was assayed by coimmunoprecipitation. The results showed that CaMKII, HDAC, MEF2 C, and atrial natriuretic peptide (ANP) expression was increased in the ISO group and downregulated by AIP treatment *in vitro*. There was no difference in the expression of these proteins between the ISO 2-week group and the ISO 3-week group *in vivo*. Ca_V_1.2 channel expression did not change, but p-Ca_V_1.2 expression was increased after ISO stimulation and decreased by AIP. In the rat model, p-Ca_V_1.2 levels and CaMKII activity were much higher in the ISO 3-week group than in the ISO 2-week group. CaMKII-induced Ca_V_1.2 channel phosphorylation at residue Thr1604 may be one of the key features of myocardial hypertrophy and disease development.**Abbreviations:** CaMKII: Ca2+/calmodulin dependent protein kinase II; p-CaMKII: autophosphorylated Ca2+/calmodulin dependent protein kinase II; CaM: calmodulin; AIP: autocamtide-2-related inhibitory peptide; ECC: excitation-contraction coupling; ISO: isoproterenol; BW: body weight; HW: heart weight; LVW: left ventricle weight; HDAC: histone deacetylase; p-HDAC: phosphorylated histone deacetylase; MEF2C: myocyte-specific enhancer factor 2C; ANP: atrial natriuretic peptide; PKC: protein kinase C

## Introduction

The Ca_V_1.2 channel is the primary source of Ca^2^+ influx, which initiates cardiac excitation-contraction coupling (ECC) [1,2]. In addition to regulating cardiomyocyte contraction, Ca^2+^ influx from the Ca_V_1.2 channel is also involved in intracellular signaling and the gene regulatory events that underlie cardiac hypertrophy and disease [3,4]. Mutations in CACNA1C, which encodes Ca_V_1.2, result in the pathogenesis of atrial fibrillation [5], ventricular fibrillation [6], hypertrophic cardiomyopathy [7] and ventricular hypertrophy [8]. Furthermore, Ca_V_1.2 is modified posttranslationally (e.g. phosphorylation) and forms a macromolecular complex [9] by binding many regulatory and helper proteins that regulate its function and expression. These interactions and posttranslational modifications may be important in acquired arrhythmias, such as those that occur in the context of hypertrophy and heart failure [10,11]. In particular, CaMKII has become known as a key regulator of Ca_V_1.2 through its phosphorylation of Ca_V_1.2 at its C-terminus [12].

CaMKII regulates the cardiac Ca_V_1.2 channel, a potential mechanism of Ca^2+^-dependent facilitation (CDF) [13]. CaMKII can regulate heart contraction and the heart rate under physiological conditions. The mutations of Ca_V_1.2 at the Ser1512 and Ser1570 phosphorylation sites in mice counteracted a potentially proarrhythmic QT interval [14]. CaMKII binding and phosphorylation sites (Thr498) on β2a subunit of Ca_V_1.2 are small but pivotal components of CaMKII-triggered cardiomyocyte death and afterdepolarizations [9]. This evidence suggests that CaMKII-dependent phosphorylation of the Ca_V_1.2 channel is essential for heart function.

In a previous study, we found that Thr1603 (Thr1603 in guinea pigs and Thr1604 in rats) is a new site at which CaMKII phosphorylates the Ca_V_1.2 C-terminus [15]. Phosphorylation of Ca_V_1.2 at Thr1603 by CaMKII can maintain the basic activity of the channel and regulate CDF. Phosphorylation of Ca_V_1.2 at Thr1603 in the pre-IQ region has been found to increase the number of Calmodulin (CaM) molecules bound to the pre-IQ-IQ region and/or enhance the interaction of CaM with pre-IQ-IQ, thereby facilitating opening of the Ca_V_1.2 channel [16]. However, the role of Ca_V_1.2 phosphorylation at the Thr1603 site (Thr1604 in rats) in heart disease remains uncertain. In this study, we focused on the CaMKII-phosphorylated Thr1604 site in the Ca_V_1.2 channel in a cardiac hypertrophy cell model and rat model.

Myocardial hypertrophy is a basic response of myocardial cells to external stimuli. Initially, myocardial hypertrophy is a beneficial adaptive response, but persistent myocardial hypertrophy caused by long-term stimulation can eventually lead to dilated cardiomyopathy, heart failure and sudden cardiac death [17,18]. Researchers have shown that the persistent stimulation of G protein-coupled β-adrenergic receptors (β-ARs) can activate CaMKII and phosphorylate histone deacetylase (HDAC) in the cytoplasm and nucleus, which releases myocyte-specific enhancer factor 2 C (MEF2)-mediated inhibition of the expression of the hypertrophic gene atrial natriuretic peptide (ANP) and eventually leads to myocardial hypertrophy [19,20]. We hypothesized that persistent activation of β-ARs could activate CaMKII and induce phosphorylation of the Ca_V_1.2 channel at Thr1604 (rat).

## Results

### ISO-induced cardiomyocyte hypertrophy in vitro

**Figure 1.**
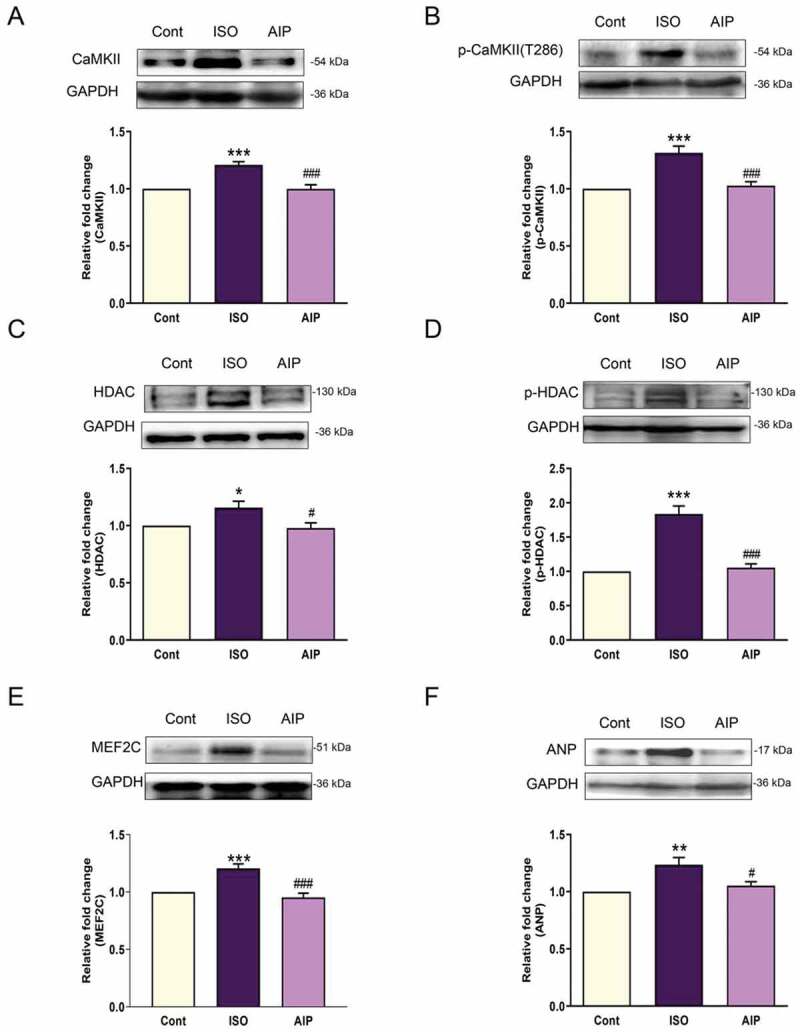
Expression of members of the CaMKII/HDAC/MEF2/ANP pathway and the effect of AIP on ISO-induced cardiac hypertrophy in a cardiomyocyte model. (a) Representative CaMKII expression and quantification. (b) p-CaMKII expression in each group. (c) HDAC expression in each group. (d) The expression of p-HDAC in each group. (e) MEF2 C expression in the cardiomyocyte hypertrophy cell model. (f) Representative ANP expression and quantification. n = 6, *P < 0.05, **P < 0.01, and ***P < 0.001, ISO group vs control group; ^#^*P* < 0.05 and ^###^*P* < 0.001, AIP group vs ISO group. AIP: autocamtide-2-related inhibitory peptide, HDAC: histone deacetylase. MEF: myocyte-specific enhancer factor.

Isoproterenol (ISO) was chosen to induce cardiomyocyte hypertrophy and activate CaMKII. Autocamtide-2-related inhibitory peptide (AIP) is a CaMKII inhibitor that inhibits the activation of CaMKII and downregulates its downstream protein expression [21]. The expression of CaMKII and the product of its autophosphorylation (p-CaMKII) was increased after ISO stimulation compared with that of the control group. AIP inhibited the ISO-induced increase in the expression of these proteins (Figure 1(a,b)). The expression levels of HDAC, p-HDAC, MEF2 C and ANP in the ISO group were all higher than those in the control group and also inhibited by AIP (Figure 1(c,d)).

### CaMKII-dependent phosphorylation of the Ca_V_1.2 channel at Thr1604 in vitro

**Figure 2.**
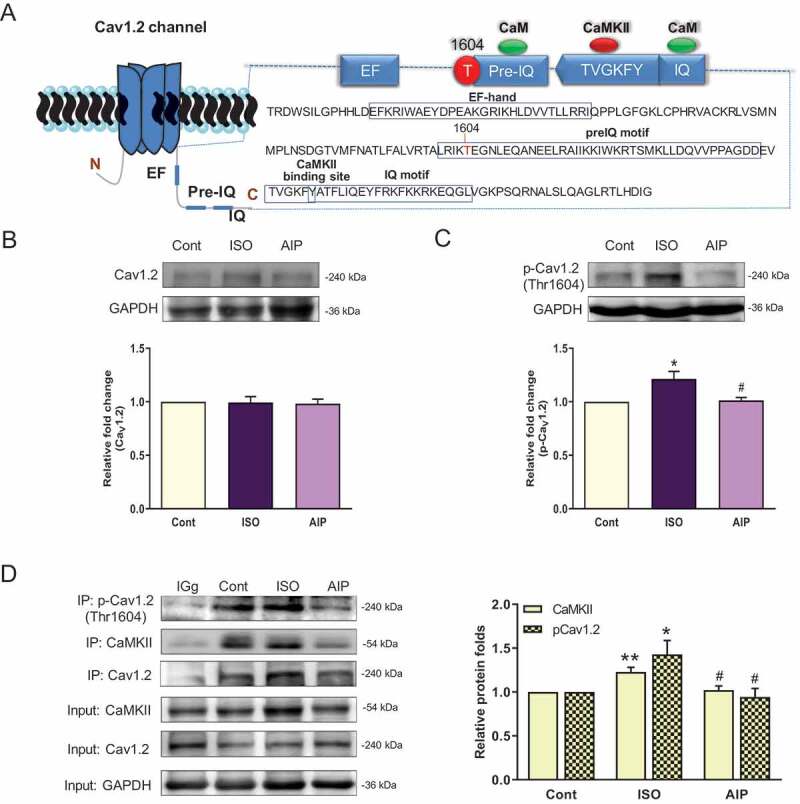
Ca_V_1.2 protein expression in an ISO-induced myocardial hypertrophy model and CaMKII-induced phosphorylation of Ca_V_1.2 (Thr1604). (a) The location of the Thr1604 CaMKII phosphorylation site on the Ca_V_1.2 channel C-terminus (GenBank: AC_000072.1, NP_036649.2 rat heart). (b) and (c) Immunoblot assay of Ca_V_1.2 and p-Ca_V_1.2 (Thr1604) protein levels in homogenates obtained from ISO-induced cultured neonatal rat cardiomyocytes. (d) Coimmunoprecipitation of p-Ca_V_1.2 (Thr1604) and CaMKII in cardiomyocytes (The bands containing CaMKII and p-Ca_V_1.2 were normalized to that of precipitated Ca_V_1.2 to measure CaMKII-induced Ca_V_1.2 phosphorylation at Thr1604.) n = 6, *P < 0.05 and **P < 0.01 vs control group, ^#^*P* < 0.05 and ^###^*P* < 0.001 AIP group vs ISO group. AIP: autocamtide-2-related inhibitory peptide.

Next, we measured Ca_V_1.2 channel protein expression *in vitro*. Western blotting showed that expression of the Ca_V_1.2 channel protein was not different among the control group, the ISO group and the AIP group. The CaMKII-phosphorylated sites on the Ca_V_1.2 channel C-terminus are shown in Figure 2(a). We used anti-p-Ca_V_1.2 (Thr1604) antibody to measure p-Ca_V_1.2 expression. Anti-p-Ca_V_1.2 (Thr1604) antibody was synthesized by Affinity Co., Ltd. The specificity of the antibody against Ca_V_1.2 phosphorylated at Thr1604 was demonstrated by western blotting. For specific experimental methods, refer to the supplementary data (Supplementary Figure 1 and Figure 2), specifically the Materials and Methods section. We found that p-Ca_V_1.2 expression was upregulated after ISO stimulation and downregulated by AIP (Figure 2(b,c)). The results of coimmunoprecipitation (Co-IP) assays showed that Ca_V_1.2 phosphorylation at Thr1604 was induced by CaMKII and increased by ISO stimulation, whereas phosphorylation of the Ca_V_1.2 channel was inhibited by AIP (Figure 2(d)). These results indicate that CaMKII-induced phosphorylation of Ca_V_1.2 at Thr1604 participates in cardiomyocyte hypertrophy.

### Pathological myocardial hypertrophy development in an ISO-induced rat model

**Figure 3.**
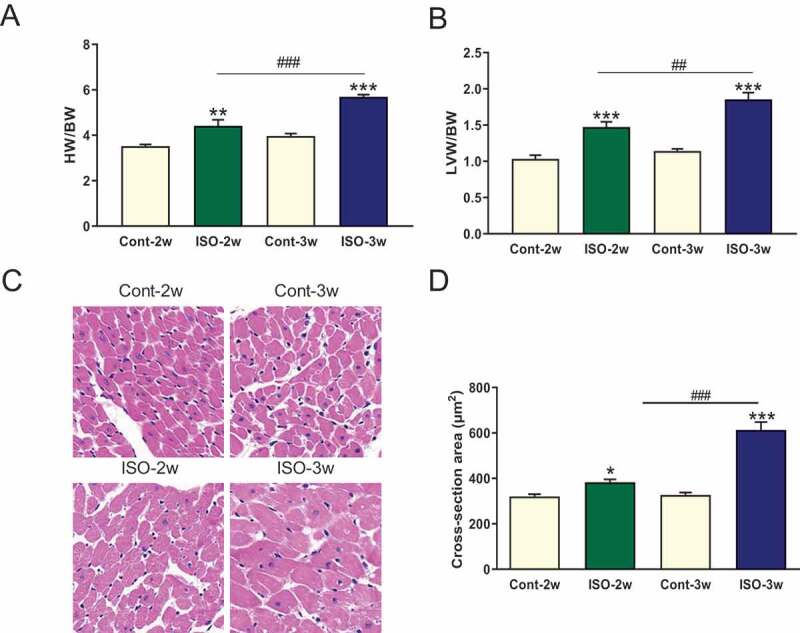
Persistent ISO stimulation induced cardiac hypertrophy in SD rats. (a) Heart weight and left ventricular weight normalized to body weight. BW: body weight, HW: heart weight, LVW: left ventricular weight. **P < 0.01 and ***P < 0.001 compared with the control group. ^##^*P* < 0.01 and ^###^*P* < 0.001 compared with the ISO 2-week group. Each group contained 6–7 rats. (b) Hematoxylin- and eosin-stained sections and cardiomyocyte cross-sectional areas in each group. Average of 10 stained sections for each heart, n = 3 hearts in each group. *P < 0.05 and ***P < 0.001 vs the control group; ^###^*P* < 0.001, ISO 2-week group vs ISO 3-week group.

Persistent β-AR stimulation has been suggested to cause myocardial hypertrophy that develops into heart failure. To observe the progression of pathological myocardial hypertrophy development, we subcutaneously injected rats with 5 mg/kg ISO for two weeks or three weeks. Myocardial hypertrophy indexes (heart weight and left ventricle weight normalized to body weight) and the cross-sectional areas of myocardial cells were used to describe myocardial hypertrophy. After ISO stimulation for two weeks, the myocardial hypertrophy indexes and cross-sectional areas of myocardial cells had increased and were much higher in the ISO 3-week group than in the ISO 2-week group (Figure 3). These results indicate that persistent ISO simulation causes the development of pathological myocardial hypertrophy.

### Role of the CaMKII/HDAC signaling pathway in cardiac hypertrophy development

**Figure 4.**
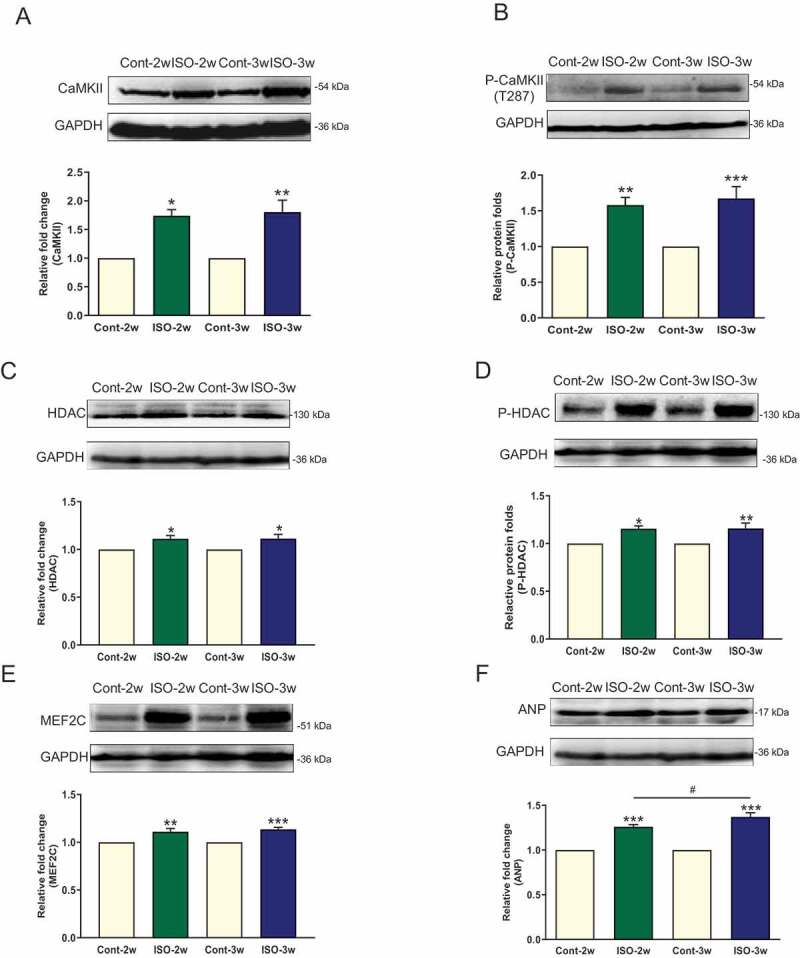
CaMKII/HDAC/MEF2/ANP expression in myocardial hypertrophy development in an SD rat model. (a) Representative CaMKII expression and quantification. (b) p-CaMKII expression in each group. (c) HDAC expression in each group. (d) The expression of p-HDAC in each group. (e) MEF2 C expression in the cardiac hypertrophy model. (f) Representative ANP expression and quantification; n = 6, *P < 0.05, **P < 0.01, and ***P < 0.001, ISO group vs control group; ^#^*P* < 0.05, ISO 2-week group vs ISO 3-week group.

We evaluated CaMKII and its phosphorylation status in the hypertrophic ventricular myocardium of model rats. After 2 and 3 weeks of ISO stimulation, the CaMKII protein level was increased at different stages of myocardial hypertrophy (Figure 4(a)). The autophosphorylation of CaMKII (producing p-CaMKII) was increased by 1.6- and 1.7-fold compared with that in the respective control group (Figure 4(b)). Although the persistent stimulation of β-AR upregulated CaMKII expression, there was no difference in the expression of CaMKII and p-CaMKII between the ISO 2-week group and the ISO 3-week group (Figure 4(a,b)). The expression levels of proteins downstream of CaMKII (HDAC, p-HDAC, and MEF2 C) were all increased in the ISO 2-week and 3-week groups, but there were no differences in their expression between the two groups (Figure 4(c,d)).

### The Thr1604 site of the rat cardiac Ca_V_1.2 channel is phosphorylated by CaMKII, which is involved in myocardial hypertrophy

**Figure 5.**
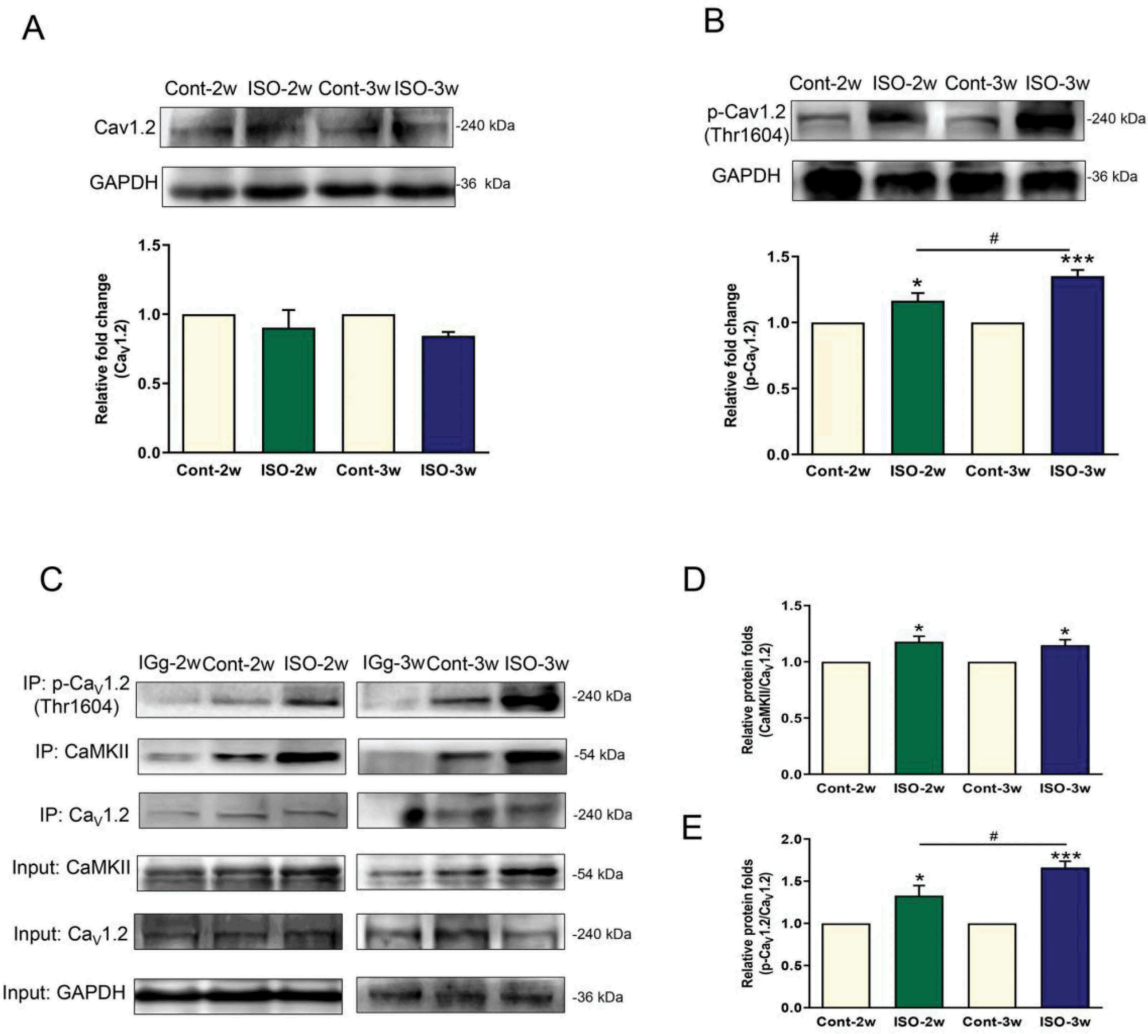
Ca_V_1.2 protein expression and CaMKII-mediated phosphorylation of Ca_V_1.2 at the Thr1604 site in the development of myocardial hypertrophy. (a) and (b) Immunoblot assay to detect the Ca_V_1.2 and p-Ca_V_1.2 (Thr1604) proteins in left ventricular homogenates obtained from ISO-induced cardiac hypertrophy rats. (C, D, E) Coimmunoprecipitation of p-Ca_V_1.2 (Thr1604) and CaMKII in homogenates. (The bands containing CaMKII and p-Ca_V_1.2 in each group were normalized to that of precipitated Ca_V_1.2 to measure the CaMKII-induced Ca_V_1.2 phosphorylation at Thr1604.) n = 6, *P < 0.05, **P < 0.01, and ***P < 0.001 vs control group, ^#^*P* < 0.05 ISO 2-week group vs ISO 3-week group.

We compared Ca_V_1.2 channel expression and p-Ca_V_1.2 channel (Thr1604) levels among the control group, the ISO 2-week group and the ISO 3-week group. The Ca_V_1.2 channel expression level did not change as cardiac hypertrophy developed, but the p-Ca_V_1.2 (Thr1604) level increased with persistent ISO stimulation and was substantially increased in the ISO 3-week group (Figure 5(a,b)). Co-IP showed that CaMKII-induced Ca_V_1.2 phosphorylation at Thr1604 was enhanced by ISO treatment. As cardiomyocyte hypertrophy worsened, the amount of CaMKII that coimmunoprecipitated with p-Ca_V_1.2 (Thr1604) was much higher in the ISO 3-week group than in the ISO 2-week group (Figure 5(c,d)).

## Discussion

Our data contribute to an increased understanding of regulatory mechanism of CaMKII and the Ca_V_1.2 channel on cardiac hypertrophy in three key areas. First, our results show that although the continuous stimulation of β-ARs can increase the expression of CaMKII and its activator, p-CaMKII, as well as the protein expression of HDAC, MEF2 C and ANP, which are downstream of CaMKII in its signaling pathway, the expression of these proteins did not change further with prolonged stimulation time. Second, there was no significant change in the protein expression of the Ca_V_1.2 channel, after continuous stimulation. Third, in an ISO-induced neonatal rat cardiomyocyte hypertrophy model and a rat model, we found that CaMKII-induced Ca_V_1.2 phosphorylation at Thr1604 increased with prolonged stimulation time.

**Figure 6.**
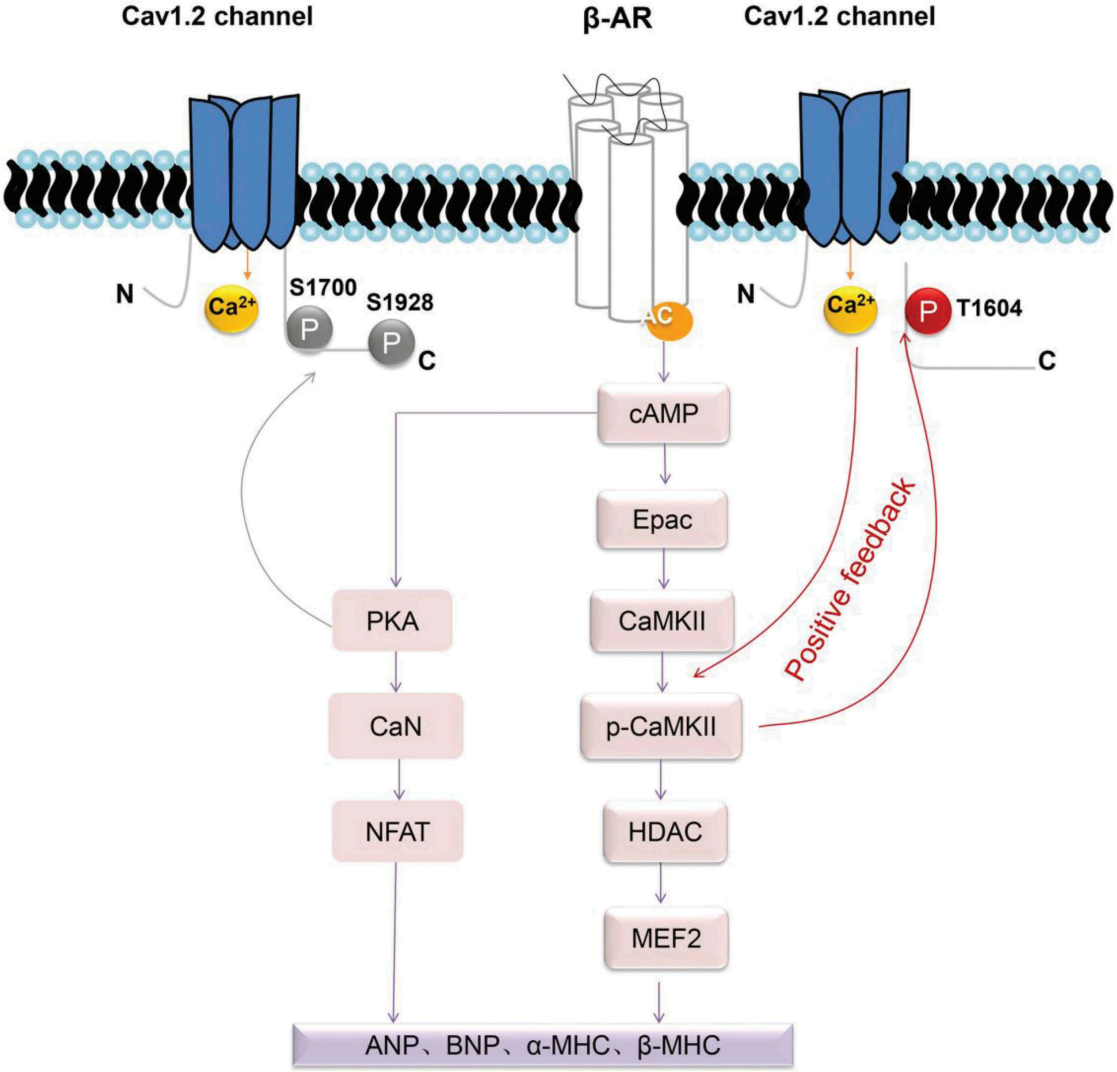
A schematic of the hypothesis that CaMKII-induced Ca_V_1.2 channel phosphorylation at the Thr1604 site induces pathological myocardial hypertrophy development in rats. Briefly, a signaling cascade after ISO-dependent stimulation of cardiomyocyte β-ARs involves the activation of Gs proteins, which in turn stimulate cyclic adenosine monophosphate (cAMP). Exchange protein directly activated by cAMP (Epac) activates CaMKII-mediated phosphorylation of Ca_V_1.2 at the C-terminal Thr1604 site, opening the Ca_V_1.2 channel and increasing intracellular calcium levels. The latter further promotes CaMKII autophosphorylation. This positive feedback mechanism enhances the CaMKII/HDAC/ANP hypertrophic signaling pathway. Moreover, elevated intracellular calcium may also act on the β-AR/PKA hypertrophic signaling pathway, as reported by the group of William A. Catterall [29,30,33], eventually leading to irreversible cardiac hypertrophy and heart failure. β-AR: β-adrenergic receptor; cAMP: cyclic adenosine monophosphate; Epac: exchange protein directly activated by cAMP; CaMKII: Ca^2+^/calmodulin-dependent protein kinase II; HDAC: histone deacetylase; PKA: protein kinase A; CaN: calcineurin; NFAT: nuclear factor of activated T cells; ANP: atrial natriuretic peptide; BNP: B-type natriuretic peptide; and β-MHC: β-myosin heavy chain.

Elevated intracellular calcium is important in myocardial hypertrophy [22]. The Ca_V_1.2 channel, the most crucial ion channel for calcium influx in cardiomyocytes, naturally participates in the process of myocardial hypertrophy and plays an important role [23–25]. A genetic reduction in the Ca_V_1.2 channel could protect the heart during stimulation by disease-causing stress, consistent with animal models treated with L-type calcium channel blockers that showed reduced hypertrophy [26–28]. In this study, our results showed that the expression level of the Ca_V_1.2 channel did not change in the myocardial hypertrophy onset group (ISO 2-week group) or in the advanced myocardial hypertrophy group (ISO 3-week group) (Figure 5(a)). However, we found that p-Ca_V_1.2 (Thr1604) expression markedly increased with the development of myocardial hypertrophy (Figure 5(b)). β-AR and Ca_V_1.2 channels are coupled in myocardial hypertrophy. Our data suggest that this coupling can be enhanced by phosphorylation of the Ca_V_1.2 channel at Thr1604, and a schematic of this mechanism is shown in Figure 6.

There are many phosphorylation sites on the cardiac Ca_V_1.2 channel protein that can regulate the physiological function of the channel [30–32]. Some of these phosphorylation sites have also been reported to be involved in heart disease, especially those activated by protein kinase A (PKA). The cardiac Ca_V_1.2 channel C-terminal domain was reported to be phosphorylated at Ser1928 and Ser1700 during β-adrenergic regulation in the heart [30,33]. Further study showed that the loss of β-adrenergic–stimulated phosphorylation of the Ca_V_1.2 channel at Ser1700 reduced contractility and hypertrophy [10]. PKA is the most widely investigated β-AR signaling molecule in the heart. However, increasing evidence has revealed that CaMKII is equally vital to the regulation of cardiac function under physiological and pathological conditions [9,12,13,34–36]. The results in this study showed that β-adrenergic-stimulated phosphorylation of the Ca_V_1.2 channel at Thr1604 is activated by CaMKII in myocardial hypertrophy (Figure 2). Thus, β-AR/CaMKII signaling and β-AR/PKA signaling coexist in hypertrophy, although the molecular mechanism remains to be elucidated. Based on these two β-AR signaling pathways, we suggest that CaMKII-mediated and PKA-mediated β-AR/Ca_V_1.2 coupling occurs in myocardial hypertrophy.

We propose the presence of a p-Ca_V_1.2 (Thr1604) positive feedback mechanism in myocardial hypertrophy (Figure 6). Briefly, activated CaMKII phosphorylates Ca_V_1.2 at its C-terminal Thr1604 site, which opens the Ca_V_1.2 channel and increases intracellular calcium levels. The latter event further promotes CaMKII autophosphorylation. This positive feedback mechanism enhances the CaMKII/HDAC/ANP hypertrophic signaling pathway. Moreover, elevated intracellular calcium may also act on the β-AR/PKA hypertrophic signaling pathway, eventually leading to irreversible myocardial hypertrophy and heart failure. A study reported that the abnormal activation of CaMKII plays a greater role in the pathological process of myocardial hypertrophy than abnormal PKA activation [37]. One possible reason for this finding is that the Ca^2+^/CaMKII/Ca_V_1.2 positive feedback mechanism is contained within the CaMKII hypertrophic signaling pathway. Clinically, β-AR antagonists can only alleviate (but not prevent) the progression of heart failure in patients with myocardial hypertrophy [38]. This p-Ca_V_1.2 (Thr1604) positive feedback mechanism can also explain this phenomenon.

Myocardial hypertrophy progresses over time [18,39] The severity of myocardial hypertrophy at 3 weeks is worse than that at 2 weeks. However, in our experiments, the expression of CaMKII and its phosphorylation level peaked at two weeks, and there was no further increase in these levels at three weeks. This is a paradox: the pathological state did not correspond with the level of CaMKII and its phosphorylation. However, we found that the phosphorylation of the Ca_V_1.2 channel increased over time. This can be seen from the results of the experiment on the phosphorylation site Thr1604. We concluded that changes in the regulatory protein CaMKII are related to the progression of myocardial hypertrophy and that phosphorylation of the Ca_V_1.2 channel at the C-terminal 1604 site mediates this progression.

In this study, we found a new CaMKII-induced phosphorylation site in the rat cardiac Ca_V_1.2 channel, Thr1604, and discovered a mechanism by which Ca_V_1.2 phosphorylation at this site participates in myocardial hypertrophy development. Further studies are needed to thoroughly understand the role of Thr1604 in the regulation of Ca_V_1.2 channel function in myocardial hypertrophy. Our study may provide a new treatment target for myocardial hypertrophy.

## Materials and methods

### Cell culture and cell model of hypertrophy

The hearts from neonatal rats (1–3 days old) were removed and digested by trypsin to obtain cardiomyocytes. Briefly, the hearts were digested, and the cells were suspended in Dulbecco’s modified Eagle’s medium (DMEM) with 20% FBS and precultured in a humidified CO_2_ incubator for 90 min to remove cardiac fibroblasts by selective adhesion. Then, the suspended cardiomyocytes were plated in another dish. The purity of the cardiomyocytes was increased by supplementation with 5-bromo-2ʹ-deoxyuridine (BrdU) to prevent noncardiomyocytes from developing. The culture medium was renewed after 48 h, and the cells were further cultured for 24 h. After the cardiomyocytes had adhered to the dish, the cardiomyocytes were divided into three groups: a control group, ISO group, and AIP treatment group. The ISO group was administered 10 μmol/L ISO for two days (48 h) [1] to induce myocardial hypertrophy. The AIP treatment group was treated with 5 μmol/L AIP [21] and 10 μmol/L ISO via coculture for 48 h. Then, the cardiomyocytes were cultured in serum-free DMEM for 12 h before experiments.

### Animal model of myocardial hypertrophy

Healthy male SD rats weighing 180–220 g (n = 36, Liaoning Changsheng Biotechnology Co., Ltd., Liaoning, China, SCXK (Liao) 2015–0001) were kept under standard conditions (temperature 21 ± 1°C; humidity 55–60%). The rats were acclimated for one week before the experiment. Food and water were freely provided to the rats. All experimental procedures followed the provisions of the Animal Protection and Use Committee of China Medical University. The rats were randomly divided into model and control groups. Rats in the model group were subcutaneously injected with 5 mg/kg/day ISO (Sigma, Switzerland) [21] for two weeks or three weeks. Rats in the control group under the same conditions were given the same volume of saline for one week, two weeks or three weeks. Each group of rats was euthanized, and the hearts were harvested and briefly rinsed in cooled PBS to remove the blood. Then, the hearts were dried and weighed. Heart weight (HW) and left ventricle weight (LVW) were normalized to body weight (BW) and used to describe myocardial hypertrophy.

### Hematoxylin and eosin staining

The SD rats were sacrificed, the hearts were removed, and the atria was removed. Slices of ventricular tissue (2 millimeters thick) were cut and fixed in 4% paraformaldehyde for 48 h. After fractional ethanol dehydration, the slices were permeabilized with xylene, and the paraffin-embedded tissues were cut into 3-µm sections. The sections were hydrated with xylene and fractional ethanol and stained with hematoxylin and eosin (HE). Cardiomyocyte cross-sectional areas were measured by ImageJ software. More than 100 cardiomyocytes were chosen from each group to analyze the cross-sectional cardiomyocyte area.

### Western blot assay

Cardiac tissues or cultured cells were homogenized in lysis buffer containing PMSF and protein kinase inhibitor. After vortexing, the lysate was centrifuged at 13,500 rpm for 15 min at 4°C and subjected to SDS-PAGE. Protein samples separated by SDS-PAGE were subsequently transferred to a polyvinylidene fluoride (PVDF) membrane, which was blocked with 5% BSA for 2 h at room temperature and incubated with the following antibodies overnight at 4°C: anti-ANP (Santa Cruz Biotechnology, sc-515,701, 1:1000), anti-GAPDH (TRANS, HC-301, 1:2000), anti-p-CaMKII (T287) (Abcam, ab171095, 1:2000), anti-CaMKII (Abcam, ab52476, 1:2000), and anti-Ca_V_1.2 (Alomone Labs, AC-003, 1:500). The membrane was washed three times and blocked with goat anti-rabbit IgG (1:10,000 dilution, EARTH) or goat anti-mouse IgG (1:10,000 dilution, EARTH) for 2 h at room temperature. ECL was used to visualize the bands. ImageJ software (http://rsb.info.nih.gov/ij/) was used for densitometry analysis.

### Co-IP assay

Protein lysates of rat hearts and cultured cardiomyocytes were preincubated with protein A-Sepharose (GE Healthcare, 17-0963-03) for 1 h at room temperature, and anti-CaV1.2 antibody (Alomone Labs, AC-003, 1:500), anti-rat or anti-rabbit IgG (Yeasen, Rat IgG, 36114ES60; rabbit IgG) were then added into the lysate overnight. IgG treatment was used as a negative control, and input proteins were used as a positive control group. On the second day, the protein-antibody-protein A-Sepharose complex was centrifuged at 1,300 rpm for 3 min at 4°C, and the supernatant was discarded. The Sepharose beads were washed with 500 μL of cold PBS three times. Proteins were extracted with 1:5 (v/v) SDS sample buffer and then centrifuged at 5,000 rpm for 3 min at 4°C. The proteins were denatured at 100°C and separated by SDS-PAGE. The remaining steps were performed as described for western blot assays. The phosphorylated Ca_V_1.2 channel was detected with anti-p-Ca_V_1.2 (Thr1604) (Affinity, 1:1000), and bound CaMKII was recognized with anti-CaMKII (Abcam, ab52476, 1:2000). Anti-p-Ca_V_1.2 (Thr1604) was generated against the C-terminal region of the p-Ca_V_1.2 (Thr1604) protein encompassing amino acid residues 1601–1614.

### Statistical analysis

All values are expressed as the mean ± SEM. Statistical analysis were performed using GraphPad Prism 5. Data were compared using one-way analysis of variance; Tukey’s post hoc test was used to determine the significance of differences between groups. Differences for which *P* < 0.05 were considered to be significant.
