# Supplementary material for: The CaMKII phosphorylation site Thr1604 in the CaV1.2 channel is involved in pathological myocardial hypertrophy in rats
Source: Channels (Austin). 2020 Apr 14;14(1):151–62. doi: 10.1080/19336950.2020.1750189 (PMC7188351; doi:10.1080/19336950.2020.1750189)
Supplement: Supplemental Material [file kchl-14-01-1750189-s001.docx]

Supplementary methods and materials

1. *cDNA construction and site-directed mutagenesis*

The proximal C-terminal fragment of guinea pig Ca_V_1.2 (GenBank AB016287) containing the Thr1603 site (CT1, amino acids 1509-1789) was cloned into the pGEX-6P-1 vector (GE Biosciences) to prepare the wild-type CT1 plasmid ^1, 2^. We compared the C-termini of the Ca_V_1.2 channels from guinea pig and rat (GenBank AC000072.1, NP036649.2 rat heart). The results showed that the Thr1603 site in the guinea pig Ca_V_1.2 channel corresponds to the Thr1604 site in the rat Ca_V_1.2 channel, and the sequence alignment is shown in Supplementary Figure 1. The CT1/pGEX-6P-1 plasmid was provided by Professor Kameyama, Kagoshima University, Japan. The rat CaMKII cDNA template was generously donated by Dr. E. Miyamoto and Dr. Y. Takeuchi of Kumamoto University in Japan. Thr1603 (Thr1604 in rats) in CT1 was mutated to an alanine residue (Thr1603Ala) to generate nonphosphorylatable CT1 (CT1-T1603A) ^2^. CaMKII was mutated into CaMKII Thr286Asp to generate autoactivated CaMKII (CaMKIIT286D) ^3^. These mutations were carried out with a QuickChange Site-Directed Mutagenesis kit (Stratagene).

2. *Preparation of GST fusion proteins*

The expression vector was transfected into the host, *E. coli* BL21 (DE3) cells. Cells were grown in LB medium at 37 ℃ until the O.D.600 was 0.6-1.0, following which protein expression was induced with 1 mM isopropyl-1-thio-b-D-galactopyranoside, and the cells were cultured at 37 ℃ for 4 h and harvested. The corresponding peptides were expressed in the form of glutathione-S-transferase (GST) fusion proteins and purified by glutathione Sepharose 4B beads (GS-4B; GE Healthcare) ^2^. The CT1-1603T and CT1-1603A peptides were immobilized on the GS-4B beads. CaMKII and CaMKIIT286D were expressed as GST fusion proteins, and the GST region was cleaved with PreScission^TM^ protease (GE Healthcare) ^2^.

3. *CaMKII- and CaMKIIT286D-mediated CT1 phosphorylation*

The precipitated complex containing immobilized GST fusion proteins (CT1-1603D or CT1-1603A) was then suspended in Tris-buffer (50 mM Tris, pH 8.0, 150 mM NaCl, and 1 mM CaCl_2_). Samples were incubated with CaMKII (pretreated with Ca^2+^/CaM) or CaMKIIT286D. Phosphorylation was initiated by 1 mM Mg^2+^-ATP and terminated after 30 minutes ^2^. The proteins were separated on an 8% SDS-PAGE gel. Phosphorylation was detected by western blotting with anti-p-Ca_V_1.2 (Thr1604) and anti-Ca_V_1.2 antibodies.

Supplementary Figure


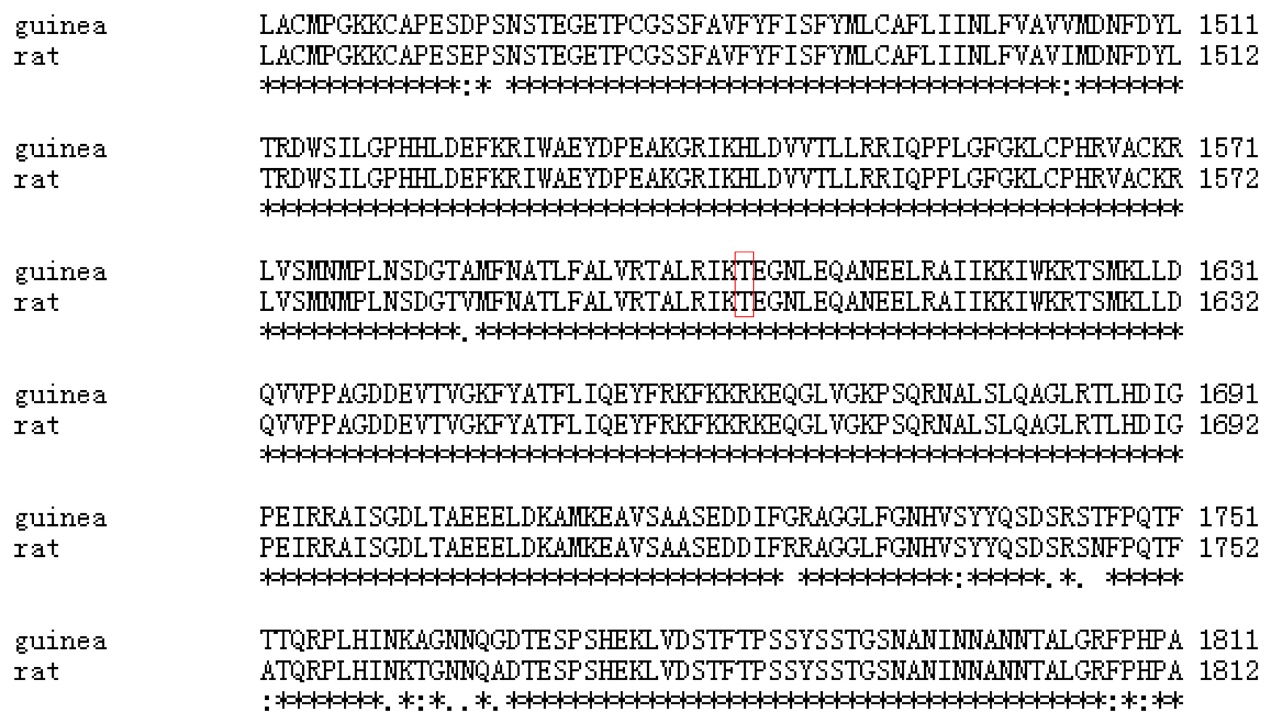


Supplementary Figure 1. BLAST alignment of the amino acid sequences of the C-termini of the Ca_V_1.2 channels from the guinea pig heart and rat heart. The amino acid in the red box is the site of the Ca_V_1.2 channel mutation (Thr1603 in guinea pig and Thr1604 in rat).


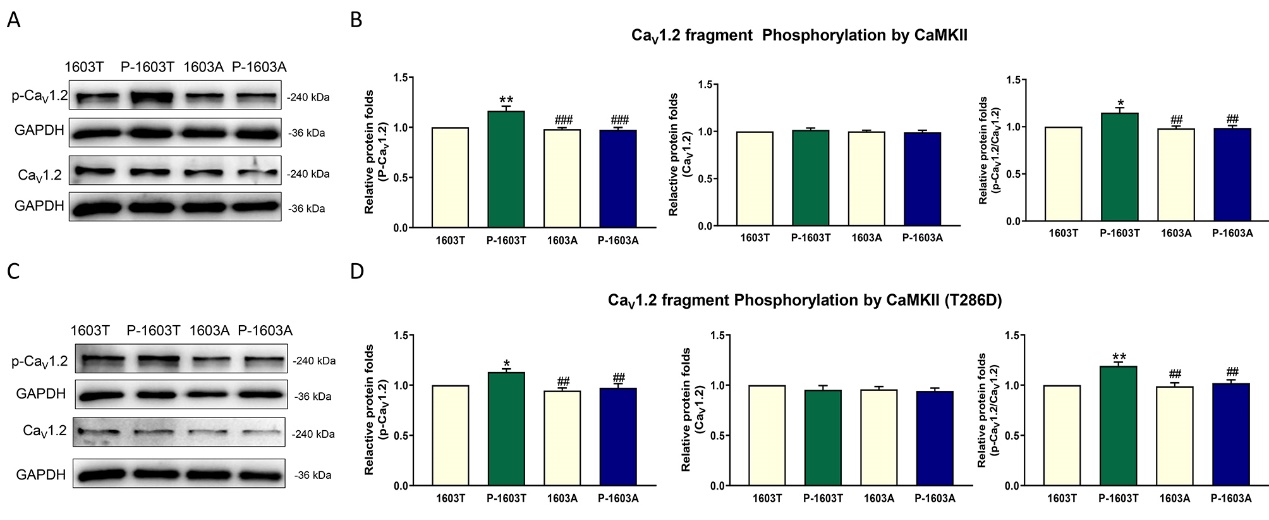


Supplementary Figure 2. Detection of the specificity of the Thr1604 phospho-AB for C-terminal peptides of Ca_V_1.2 expressed by the *E. coli* BL21 host. A and B, CaMKII-mediated phosphorylation of GST-fused CT1 (1630T) and Thr1603Ala mutant CT1 (1603A) was detected by immunoblot analysis using anti-phospho-Ca_V_1.2 (Thr1604). C and D, Immunoblot analysis of CaMKIIT286D-mediated phosphorylation of CT1 (1630Thr) and The1603Ala mutant (1603A) CT1 in *E. coli* BL21. n=5 in each group, ^*^*P*<0.05, ^**^*P*<0.01 vs the 1603Thr group, ^##^*P*<0.01, ^###^*P*<0.001 vs p-1603Thr.

References

1. Ding S, Kuroki S, Kameyama A, Yoshimura A, Kameyama M. Cloning and expression of the Ca2+ channel alpha1C and beta2a subunits from guinea pig heart. Journal of biochemistry 1999; 125:750-9.

2. Wang WY, Hao LY, Minobe E, Saud ZA, Han DY, Kameyama M. CaMKII phosphorylates a threonine residue in the C-terminal tail of Cav1.2 Ca(2+) channel and modulates the interaction of the channel with calmodulin. Journal of Physiological Sciences 2009; 59:283-90.

3. Hao LY, Wang WY, Minobe E, Han DY, Xu JJ, Kameyama A, et al. The distinct roles of calmodulin and calmodulin kinase II in the reversal of run-down of L-type Ca(2+) channels in guinea-pig ventricular myocytes. Journal of pharmacological sciences 2009; 111:416-25.
